# A Mini-Patch Magnetic Insulin Pump for Enhanced Delivery Resolution and Accuracy

**DOI:** 10.1002/aisy.202500459

**Published:** 2025-08-15

**Authors:** Qiji Ze, Shuhao Huang, Yilong Chang, Jize Dai, Rayhan A. Lal, Ruike Renee Zhao

**Affiliations:** Department of Mechanical Engineering, Stanford University, Stanford, CA 94305, USA; Department of Mechanical Engineering, Stanford University, Stanford, CA 94305, USA; Department of Mechanical Engineering, Stanford University, Stanford, CA 94305, USA; Department of Mechanical Engineering, Stanford University, Stanford, CA 94305, USA; Departments of Medicine & Pediatrics, Divisions of Endocrinology, Stanford University, Stanford, CA 94305, USA; Department of Mechanical Engineering, Stanford University, Stanford, CA 94305, USA

**Keywords:** continuous subcutaneous insulin infusion, drug delivery, insulin administration, insulin pump, magnetic actuation

## Abstract

Insulin pumps typically use piston-based mechanisms with bulky transmission components to convert rotary motion into the piston’s forward motion. These mechanical transmission systems and insulin reservoirs occupy more than one-third of pumps’ volume, significantly limiting miniaturization and making pumps cumbersome for daily use. Herein, a compact, magnetically actuated insulin pump is developed that is less than one-quarter the size of piston-based pumps. Instead of bulky mechanical components, the pump uses a magnetic soft actuator to directly compress the insulin chamber, controlled by a precisely tuned electromagnetic field. This innovative design eliminates the need for large transmission systems, enabling a notably smaller form factor. In addition, the fine-tunable magnetic actuation enables a 0.01 μL delivery resolution, significantly surpassing the 0.25 μL resolution of piston-based pumps. This high-resolution mechanism facilitates further miniaturization by allowing the use of high-concentration insulins, thereby reducing the reservoir size. By varying the magnetic field’s waveform, amplitude, and duration, the pump’s performance can be further enhanced. The reported magnetic insulin pump exhibits superior repeatability and accuracy across single-pulse, basal, and bolus modes compared to commercial insulin pumps. This miniaturized, high-resolution magnetic insulin pump is anticipated to substantially benefit people with diabetes by improving portability, precision, and cost efficiency.

## 1. Introduction

Diabetes is a chronic disease caused by an imbalance in blood glucose regulation, typically due to insufficient insulin production or the body’s inability to effectively use insulin.^[1]^ Today, it is one of the most prevalent and serious chronic diseases worldwide, affecting over 500 million people, with the number increasing by about 3%–5% every year.^[2,3]^ For people living with diabetes who need insulin replacement, multiple daily injection (MDI) insulin therapy and continuous subcutaneous insulin infusion (CSII) are two common approaches to insulin delivery.^[4]^ MDI typically requires at least four daily injections, which presents several challenges: increased burden, a higher risk of injection site infections, inconsistent blood glucose control, and potentially severe complications.^[5,6]^ CSII, the insulin therapy delivered by insulin pumps, offers several advantages over MDI: 1) the continuous insulin delivery ensures more stable glucose levels with fewer fluctuations, 2) the automated dosing provides greater accuracy than manual insulin injections, and 3) the reduced complexity in insulin administration alleviates burden and enhances quality of life.^[7−9]^

**Figure 1.**
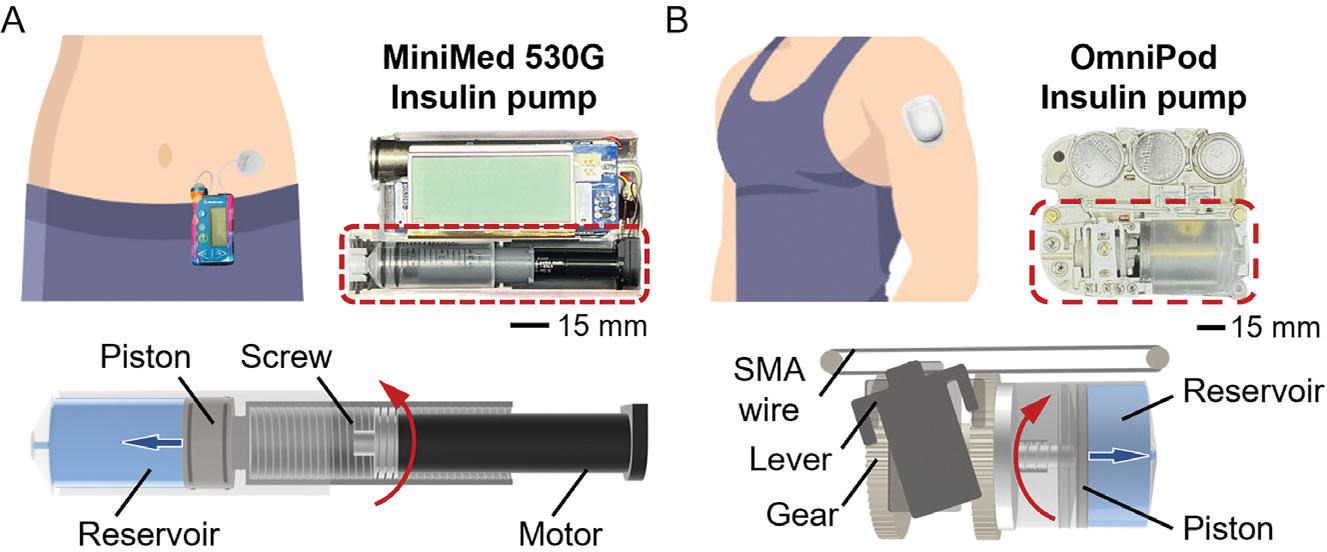
Pumping mechanisms of two commercial insulin pumps. A) MiniMed 530 G insulin pump (Medtronic Ltd, United States). B) OmniPod insulin pump (Insulet Corporation, United States).

An insulin pump usually consists of several necessary components, including the pumping mechanism, insulin reservoir, controller, and battery. Despite the diverse mechanical or nonmechanical pumping mechanisms proposed for drug delivery applications,^[10,11]^ most commercial insulin pumps still rely on piston-based mechanisms,^[12,13]^ owing to their relatively high technological maturity and insulin delivery accuracy. Taking the Medtronic MiniMed 530 G insulin pump (Figure 1A) as an example, the pumping mechanism involves a small motor that pushes a piston forward in tiny increments, forcing insulin through the tubing and into the infusion set.^[14]^ This process is enabled by the long force transmission systems, which convert the motor’s rotary motion to the piston’s forward motion through screws. These bulky mechanical transmission systems, along with the integrated insulin reservoir, occupy 28.27 cm^3^, which is approximately one-third of the pump’s total volume. Another widely used insulin pump, the OmniPod (Figure 1B), uses a thin shape memory alloy (SMA) wire as an actuator for insulin delivery.^[15,16]^ The SMA wire periodically retracts and releases a connecting lever, generating a rotary reciprocating motion. The motion is then converted into the piston’s forward movement through a set of intricate gears. Despite the OmniPod’s smaller pumping mechanism compared to the MiniMed, the necessary mechanical transmission components and integrated insulin reservoir still occupy 11.14 cm^3^, ≈40% of the device’s total volume. These complex mechanical systems significantly hinder further miniaturization, making wearing the pump inconvenient for daily activities.

Current insulin pumps are optimized for the delivery of commonly available U-100 insulin in the adult dosing range. To ensure at least 2–3 days of insulin supply, the insulin reservoir size is between 2 mL (200 units U-100) for the OmniPod and 3 mL (300 units U-100) for the MiniMed 530 G pump. With greater development and availability of more concentrated insulin formulations, it is becoming feasible to reduce the reservoir size, facilitating further miniaturization of insulin pumps.^[17,18]^ This approach necessitates a higher delivery resolution to precisely control smaller insulin doses to ensure accurate dispensing. Increasing the delivery resolution is also crucial for individuals with high insulin sensitivity or low insulin requirements, such as infants and children,^[19,20]^ to ensure precise and safe insulin dosing. However, the delivery resolution of the existing pumps is restricted by the minimum step size of screws and gears in mechanical transmission systems, making the precise delivery of high-concentration insulin challenging and further hindering the potential for pump miniaturization.

**Figure 2.**
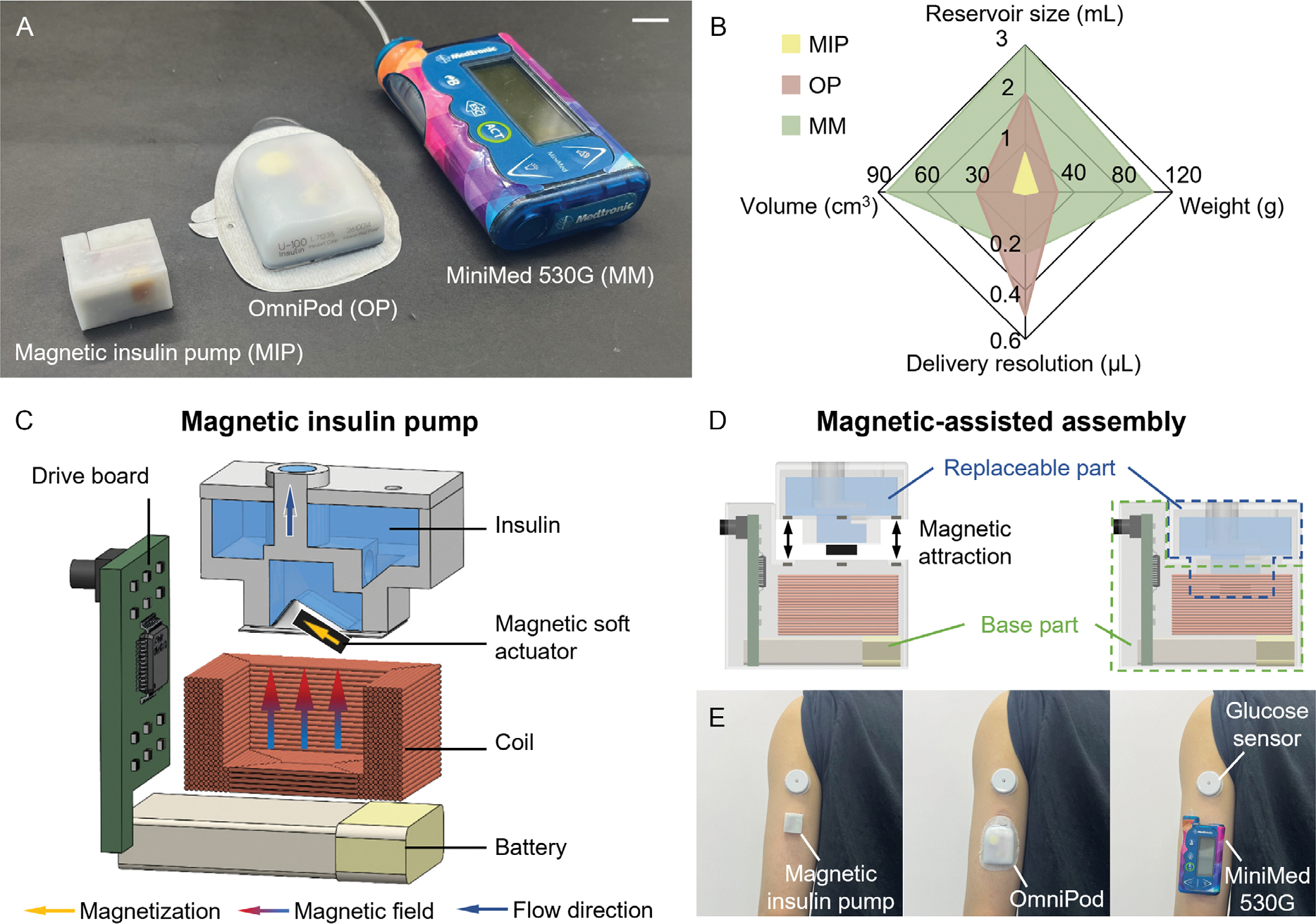
Magnetic insulin pump design. A) The magnetic insulin pump and two commercial insulin pumps. Scale bar: 10 mm. B) Comparison of the magnetic insulin pump with OmniPod and MiniMed on volume, reservoir size, weight, and delivery resolution. C) Schematic of the magnetic insulin pump. The pumping mechanism utilizes a magnetic soft actuator, which directly compresses the insulin chamber for delivery. Controlled by a magnetic field, the actuator undergoes continuous deformation, enabling finely adjustable delivery resolution. D) Magnetic-assisted assembly. The pump consists of two magnetically assembled parts. The base part, which houses the battery, driver board, and electromagnetic coil, and the replaceable part, which contains the reservoir, chamber, and magnetic soft actuator. E) On-body demonstrations of insulin pumps with FreeStyle Libre glucose sensors (Abbott Diabetes Care Ltd, UK). The system using the magnetic insulin pump offers improved portability compared to those with OmniPod and MiniMed.

In this work, we develop a compact, magnetically actuated insulin pump with high delivery resolution. The device occupies less than one-quarter the size of conventional piston-based pumps (Figure 2A). As shown in Figure 2B and Table S1, Supporting Information, the magnetic insulin pump has a volume of 7.07 cm^3^, significantly smaller than the MiniMed (84.74 cm^3^) and OmniPod (29.39 cm^3^). Meanwhile, it only weighs 10.5 g, which is also much lighter than MiniMed (104 g) and OmniPod (26 g). The miniaturization is enabled by a unique magnetic pumping mechanism, where insulin dispensing is achieved through a magnetic soft actuator that directly compresses the insulin chamber. This process is controlled by a precisely generated magnetic field from an embedded electromagnetic coil within the pump (Figure 2C, see Video S1 in the Supporting Information for the controllable liquid delivery of the magnetic insulin pump). By eliminating the bulky force transmission components required in piston-based pumps, this design significantly reduces the space occupied by the pumping mechanism to just 1.63 cm^3^, far smaller than that of MiniMed and OmniPod. In addition, the fine-tunable magnetic actuation enables high-resolution insulin delivery (Figure 2B), achieving a delivery resolution of 0.01 μL (0.001 units U-100 insulin), which is a significant improvement over MiniMed’s 0.25 μL (0.025 units U-100 insulin) and OmniPod’s 0.5 μL (0.05 units U-100 insulin). This high resolution allows for further pump miniaturization by enabling the use of high-concentration insulins to reduce the insulin reservoir size to only 0.8 mL (Figure 2B). In this way, the compact reservoir can store 80 units of U-100 insulin, 160 units of U-200 insulin, or hypothetically 400 units of a rapid-acting U-500 insulin. For individuals using less than 57 units of insulin per day, the 0.8 mL of hypothetical U-500 insulin could provide enough insulin for one week. Beyond its enhanced delivery resolution, we further experimentally demonstrate that the reported magnetic insulin pump exhibits superior repeatability and accuracy compared to MiniMed and OmniPod across single-pulse, basal, and bolus delivery modes.

Additionally, the magnetic insulin pump is designed as a two-part system assembled via magnetic attraction (Figure 2D, see Figure S1 and Video S1, Supporting Information), for the working principle and demonstration of the magnetic-assisted assembly). This system consists of a base part and a replaceable part. The base part houses the battery, driver board, and electromagnetic coil, while the replaceable part contains the reservoir, chamber, and magnetic soft actuator. This modular design enables quick and convenient insulin replacement. At the same time, replacing an OmniPod every 3 days means throwing away batteries, microcontrollers, and plastics, which carries environmental impact. Since the magnetic insulin pump’s base part is reusable, its overall usage cost and environmental sustainability have improved as well. Figure 2E shows the on-body demonstrations of insulin pumps with glucose sensors. Due to the compact design of the magnetic insulin pump, the system utilizing it offers superior wearability compared to those with OmniPod and MiniMed. Next, the magnetic insulin pump can incorporate biocompatible adhesives similar to those widely used in commercial insulin pumps, thereby ensuring secure skin attachment and proven skin-contact safety. We anticipate this miniaturized, high-resolution magnetic insulin pump to enhance the user experience and deliver significant benefits to people with diabetes by improving portability, precision, and cost efficiency.

## 2. Results

### 2.1. Working Mechanism of the Magnetic Insulin Pump

**Figure 3.**
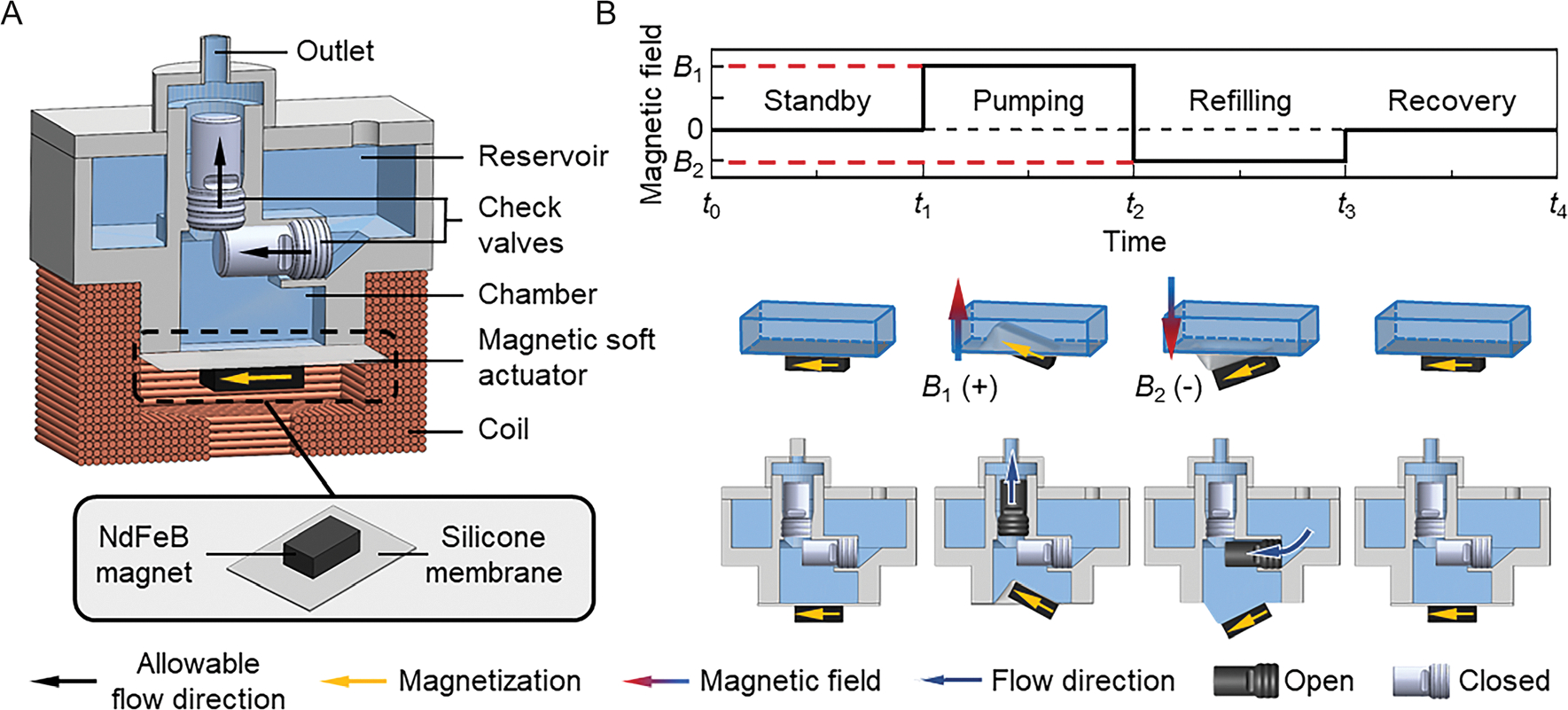
The working mechanism of the magnetic insulin pump. A) Isometric view of the magnetic insulin pump. The inset shows the composition of the magnetic soft actuator. B) Cyclic actuation of the pump: When the positive magnetic field *B*_1_ is applied, the magnetic soft actuator deflects upward, pushing the liquid out through the top one-way check valve. When a negative magnetic field *B*_2_ is applied, the magnetic soft actuator deflects downward, allowing the liquid in the reservoir to refill the insulin chamber through the bottom check valve. Once the magnetic field is removed, both check valves close. The pump then takes a short recovery period to restore pressure equilibrium before the next actuation cycle.

Magnetic actuation^[21,22]^ has been widely explored as a pumping mechanism for both wearable^[23,24]^ and implantable^[25,26]^ micropumps due to its advantages, including wireless operation, flexible control, and simplified design. Most existing micropumps rely on the magnetic attraction force^[27,28]^ for actuation. Determining the optimal distance between the magnetic field source and the actuator is challenging, as it requires balancing actuation efficiency and control precision.^[29,30]^ This constraint limits the compactness of insulin pump designs, making it difficult to optimize performance while maintaining a small form factor. To overcome these challenges, we design the proposed magnetic insulin pump, whose operation is based on magnetic torque,^[31,32]^ which allows for both structural compactness and precise control. As shown in the isometric view of the assembled insulin pump (Figure 3A), the magnetic soft actuator is in direct contact with the insulin chamber and is surrounded by the electromagnetic coil, effectively minimizing the space required for magnetic actuation components. This magnetic torque-based actuation offers a more stable control mechanism over magnetic attraction-based actuation. In this approach, the rate of torque increases gradually with small increments as the magnetic field strengthens, significantly improving control precision and enabling more accurate insulin delivery.

The magnetic pumping mechanism operates through the magnetic soft actuator, which compresses the insulin chamber when a precisely controlled magnetic field is generated by the electromagnetic coil. The magnetic soft actuator (Figure 3A) is constructed by attaching an NdFeB magnet with longitudinal magnetization (N52, Dimension: 4.0 × 2.3 × 1.4 mm) to the thin silicone membrane (Ecoflex 00–30, Thickness: 0.25 mm) that is used to seal the insulin chamber (see “Experimental Section” for the detailed fabrication process of the magnetic insulin pump). By applying electric currents with tunable amplitudes and directions to the electromagnetic coil, a controllable magnetic field along the coil’s axial direction is generated, inducing magnetic torque in the NdFeB magnet (see “Supplementary Methods” and Figure S2, Supporting Information, for the detailed information of the electromagnetic actuation system). The magnetic torque is calculated as T=V(M×B), where V is the volume of the actuator, M is the magnetization of the magnet, and B is the magnetic field acting on the magnet. Here, the amplitude of the magnetic field is defined as positive when the magnetic field direction is upwards. All magnetic field amplitudes mentioned in this article specifically refer to that at the center position of the magnet in the initial state of the magnetic soft actuator. As shown in the middle part of Figure 3B, when the magnetic field is positive, the magnet presses the membrane to reduce the chamber volume for insulin delivery. Conversely, when the magnetic field is negative, the magnet pulls out the membrane to refill the insulin chamber (see “Supplementary Methods”, Figure S3 and S4, Supporting Information, for the verification of the magnetic actuation mechanism).

The actuation process of the magnetic insulin pump consists of four sequential states, as illustrated in the bottom part of Figure 3B (see Video S2 in the Supporting Information for the actuation process of the magnetic insulin pump). By sequentially applying positive and negative magnetic fields, the pump enables the pumping and refilling of liquid, assisted by two one-way check valves (cracking pressure: 4 kPa, see Figure S5, Supporting Information, for the schematic of the check valve). These valves regulate the flow direction of the insulin, ensuring controlled delivery and refill during each cycle. The top valve connects the chamber and the pump outlet, and it only allows flow to go from the chamber to the pump outlet, while the bottom valve connects the chamber and the reservoir, only allowing the flow to go from the reservoir to the chamber. In the standby state, both check valves remain closed. The sequence of operations progresses as follows. 1) Pumping state: At time *t*_1_, a positive magnetic field *B*_1_ is applied, causing the magnetic soft actuator to press the insulin chamber, gradually increasing the pressure. When the liquid pressure difference between the chamber and the pump outlet exceeds the cracking pressure of the top check valve, the valve opens, allowing the fluid to be pumped out. At the same time, the bottom check valve remains. 2) Refilling state: At time *t*_2_, a negative magnetic field *B*_2_ is applied, causing the magnetic soft actuator to pull out the insulin chamber membrane. The pressure in the chamber quickly decreases, which helps close the top check valve and open the bottom check valve. This allows the liquid from the reservoir to refill the chamber through the bottom check valve. 3) Recovery state: After refilling, the pump enters the recovery state. When the magnetic field is removed at time *t*_3_, the magnetic soft actuator returns to its initial position. The pump requires a short period to ensure that both check valves are closed and to recover pressure equilibrium, awaiting the next actuation cycle. By precisely adjusting the magnetic field profile, the proposed magnetic insulin pump enables controllable and repeatable liquid delivery, ensuring accurate and reliable insulin administration.

### 2.2. Finite Element Analysis for Design Optimization

To achieve the best delivery efficiency, finite element analysis is performed to optimize the design parameters of the electromagnetic coil and the magnetic soft actuator in the magnetic insulin pump. Here, we preset the dimensions of the coil and the membrane shown in (Figure S6, Supporting Information) to simplify the optimization process. The effective region of the silicone membrane is 6.5 mm × 4.0 mm. Only the diameter of the copper wire in the coil and the distance from the magnet to the short edge of the membrane are taken as variables for optimization. The optimization objective is to achieve maximum magnetic field output and volume change of the magnetic soft actuator (see “Supplementary Methods” in the Supporting Information for more details on the optimization of the electromagnetic coil and magnetic soft actuator).

**Figure 4.**
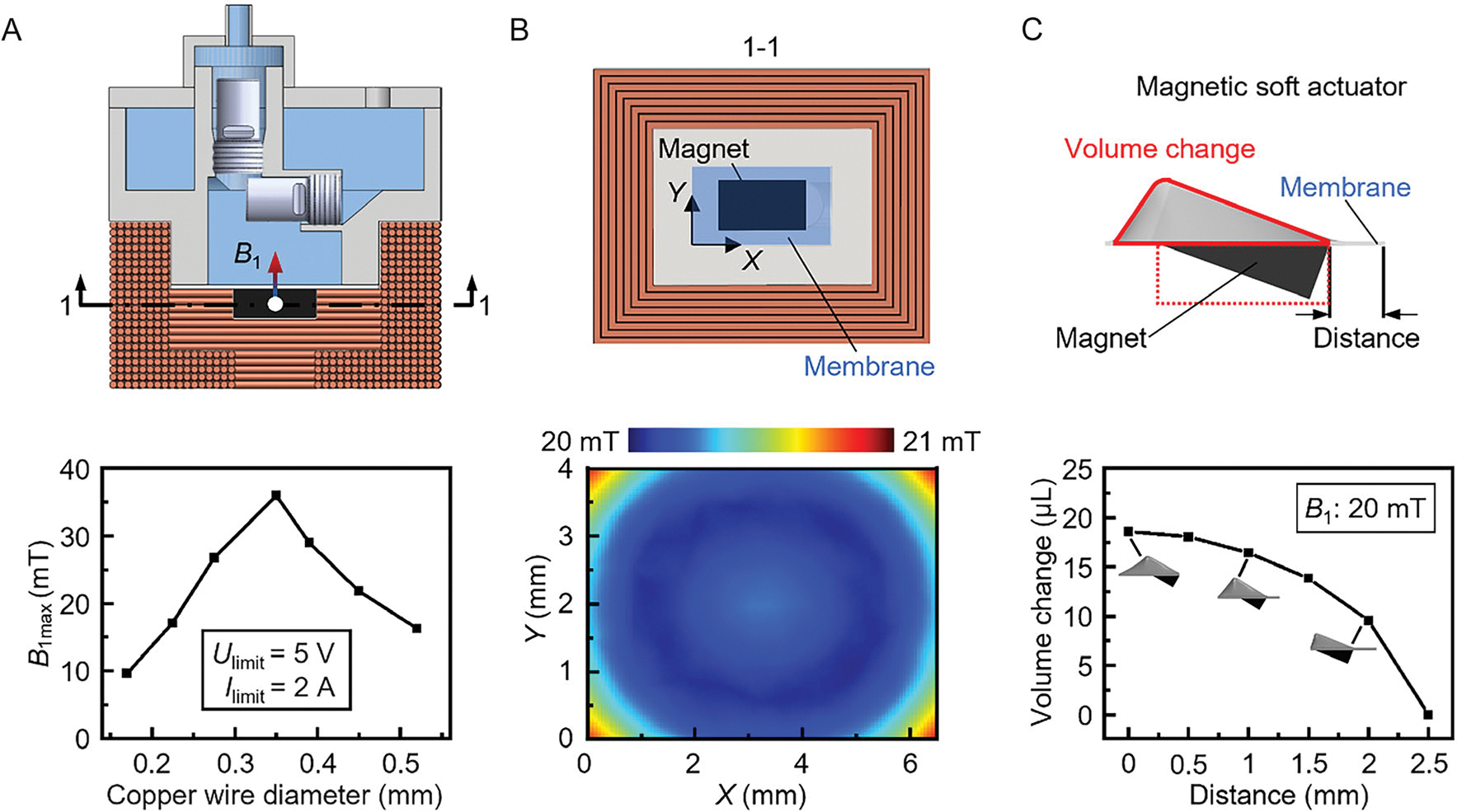
Finite element analysis for magnetic insulin pump design optimization. A) Simulation results of the magnetic field strength at the center position of the magnet for different copper wire diameters. The output voltage and current of the pump’s driver board are limited to 5 V and 2 A, respectively. B) Simulated magnetic field distribution across the membrane. C) Simulation results of the volume change of the magnetic soft actuator at varying distances from the magnet to the right short edge of the membrane.

Under the given dimensional constraints, changing the diameter of the copper wire affects both the resistance and the number of turns in the coil, thereby directly impacting the achievable magnetic field output. It is also worth noting that, considering the discharge capability of the battery, the output voltage and current of the pump’s driver board are limited to 5 V and 2 A, respectively (see “Supplementary Methods” in the Supporting Information for the thermal safety evaluation of the magnetic insulin pump). In this case, it can be seen from the simulation results in Figure 4A that the coil can generate a maximum magnetic field of 36 mT when the copper wire diameter is 0.35 mm. Thus, the diameter of 0.35 mm is used in the winding of the coil to guarantee the output capability of the electromagnetic coil. Taking the unavoidable gap between adjacent copper wires into account, the actual number of turns and resultant resistance in the coil are 206 and 2.02 ohms, respectively.

Beyond coil parameters, the relative positioning of the magnet and the membrane within the magnetic soft actuator plays a crucial role in the delivery performance of the magnetic insulin pump. The membrane deformation directly correlates with the chamber pressure during both the pumping and refilling states. As shown in Figure 4B, the magnetic field generated by the coil remains relatively uniform across the membrane (*X*-*Y* plane), with an amplitude variation of less than 5% (see Figure S7, Supporting Information, for the magnetic field distribution on the *X*-*Z* plane). Consequently, the magnet produces approximately the same actuation force on the membrane regardless of its distance from the membrane’s edge. To further investigate the effect of relative positioning, parametric simulations are conducted to study the membrane deformation. As illustrated in Figure 4C, the volume change of the magnetic soft actuator due to the deformed membrane decreases as the distance between the magnet and the right short edge of the membrane increases. To maximize delivery efficiency, the design aligns the right short edges of both the magnet and the membrane to ensure the optimal insulin delivery of the magnetic insulin pump.

### 2.3. Insulin Delivery under Different Magnetic Field Profiles

**Figure 5.**
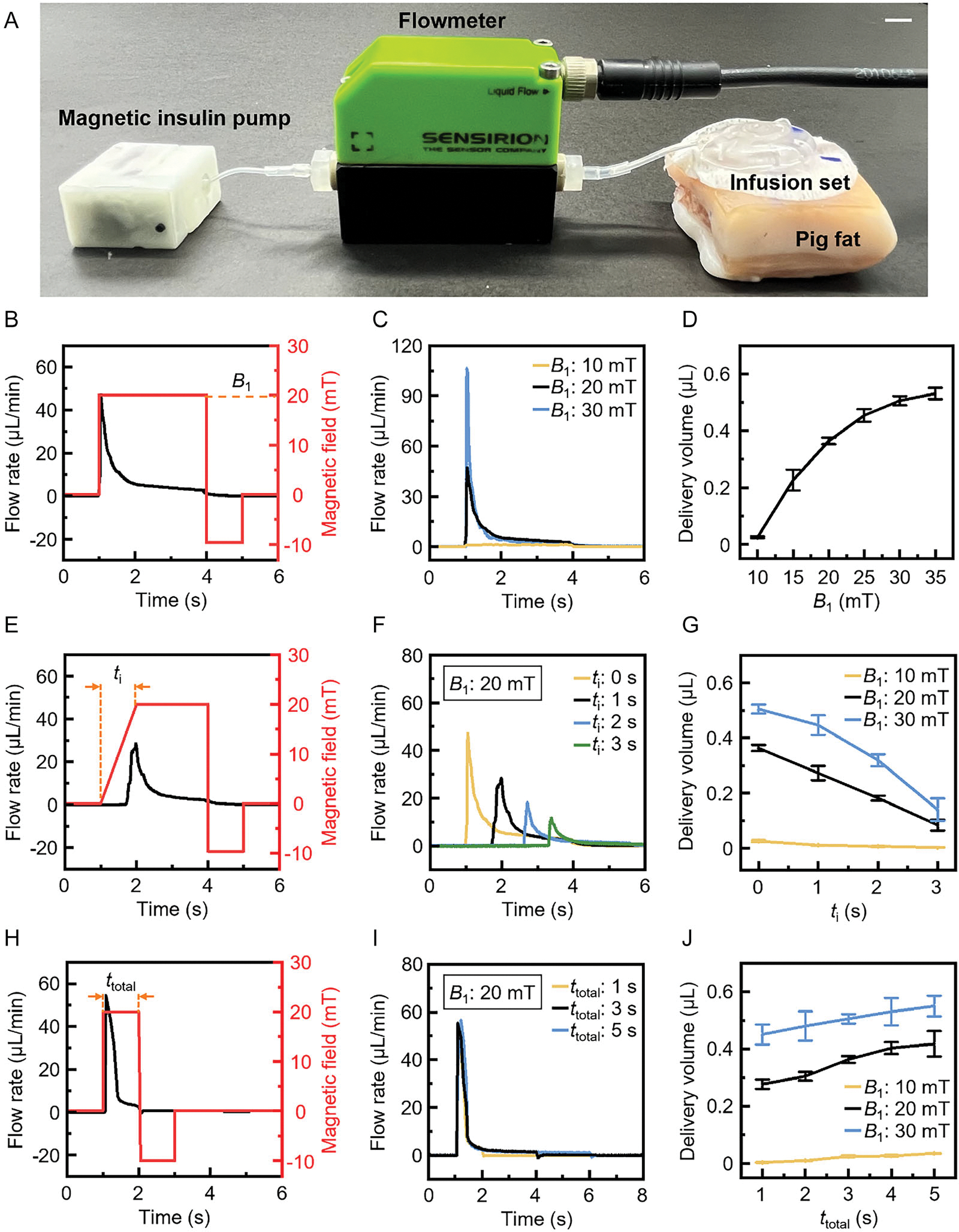
Characterization of the magnetic insulin pump under various magnetic field profiles. A) Experimental setup. Scale bar: 5 mm. B) Extrusion flow rate with a square-wave magnetic field (20 mT, 3 s duration). C) Flow rates and D) delivery volumes for square-wave magnetic fields of varying amplitudes. E) Extrusion flow rate with a trapezoidal-wave magnetic field (20 mT, 3 s duration, 1 s ramp-up time). F) Flow rates for trapezoidal-wave magnetic fields with varying ramp-up times at 20 mT. G) Delivery volumes for trapezoidal-wave magnetic fields with varying ramp-up times and amplitudes. H) Extrusion flow rate with a square-wave magnetic field (20 mT, 1 s duration). I) Flow rates for square-wave magnetic fields with varying durations at 20 mT. J) Delivery volumes for square-wave magnetic fields with varying durations and amplitudes.

The delivery accuracy of the magnetic insulin pump depends on its delivery rate, which can be actively adjusted by modifying the magnetic field profiles. To optimize the pump’s delivery performance, we conduct a systematic analysis on how different magnetic field parameters influence the pump’s ability to deliver precise and tunable doses, ensuring robust performance across diverse operating conditions and providing insights for clinical applications. Key parameters include the waveform, amplitude, and application duration of the magnetic field, which can be controlled by applying programed current to the electromagnetic coil. To evaluate the pump performance, a high-precision flow meter (SLI-0430, Sensirion, Switzerland) is used to measure the instantaneous flow rate and accumulated delivery volume. Figure 5A shows the experimental setup (see Video S2 in the Supporting Information for the demonstration of the experimental procedure). The pump, flow meter, and infusion set are connected through silicone tubing. The needle (diameter: 0.37 mm, length: 6 mm) on the infusion set is inserted through the pork skin into the fat to mimic the actual use of an insulin pump. We first optimize the parameters of refilling and recovery states to ensure effective operation. Based on the test results, the refilling magnetic field is set as −10 mT for 1 s, with a 20 s waiting time to allow both check valves to close and restore pressure equilibrium (see “Supplementary Methods” in the Supporting Information for more details on magnetic actuation experiments of the magnetic insulin pump).

Next, the influence of the magnetic field of the pumping state on drug delivery performance is systematically studied. As shown in Figure 5B–D, square-wave pumping magnetic fields with varying amplitudes and a fixed duration of 3 s are evaluated. In Figure 5B, a 20 mT pumping magnetic field reaches its target value within 0.1 s (Figure S8A, Supporting Information). The rapid pressure increase in the chamber causes a peak in the extrusion flow rate, which stabilizes gradually. When the magnetic field shifts from 20 to −10 mT at 4 s, the liquid pressure drops sharply, causing an immediate halt in flow. Since the magnetic field amplitude directly affects the deformation of the chamber membrane, adjusting it allows for precise control over the extrusion flow rate and delivery volume. As shown in Figure 5C,D, liquid extrusion begins at 10 mT, with a peak flow rate of ≈0.45 μL min^−1^ and a delivery volume of 0.023 μL per cycle. As the amplitude increases, both values rise. At 20 and 30 mT, the delivery volumes per cycle are 0.364 and 0.506 μL, respectively, demonstrating that the pump’s output can be finely tuned over a relatively wide range by controlling the magnetic field amplitude (see Figure S9, Supporting Information for complete measurement results).

Next, trapezoidal-wave pumping magnetic fields with varying ramp-up times and amplitudes and a fixed duration of 3 s are applied to assess the influence of waveform on pump performance, as shown in Figure 5E–G. In Figure 5E, the ramp-up time of the trapezoidal wave is 1 s (see Figure S8B, Supporting Information, for the actual waveform), and the corresponding peak flow rate is about 28.35 μL min^−1^, which is lower than that with a square-wave magnetic field. Furthermore, the peak flow rate gradually decreases as the ramp-up time increases (Figure 5F), resulting in decreased delivery volume per pumping cycle (Figure 5G, see Figure S10, Supporting Information, for complete measurement results). More importantly, the delivery volume can be easily tuned to be less than 0.01 μL, meaning a delivery resolution higher than 0.01 μL is achievable, which shows substantial improvement over current insulin pump products.

Finally, square-wave pumping magnetic fields with different application durations are evaluated, as shown in Figure 5H–J. When the duration changes from 1 to 5 s, the flow rate curves almost overlap except for the zero-crossing points (Figure 5I). Since the stable flow rate is very low, increasing the duration does not significantly raise the delivery volume per pumping cycle (Figure 5J, see Figure S11, Supporting Information, for complete measurement results). Based on the calculated results of delivery volume per joule in (Figure S12, Supporting Information), the magnetic field amplitude and duration should not be too large to improve energy efficiency in practical use. The delivery volume per joule reaches its maximum when the magnetic field amplitude and duration are set to 20 mT and 1 s, respectively. Under these conditions, the magnetic insulin pump delivers ≈0.1 μL of liquid per joule of energy consumed, which corresponds to 0.05 units of insulin when hypothetically using U-500 insulin. The rechargeable 120 mAh single-cell lithium-ion battery can support the delivery of ≈80 units of insulin, which is sufficient to meet the estimated two-day insulin requirement for an adult with diabetes. It is worth noting that the current single-cell battery has a compact dimension of only 20 × 10 × 4 mm (0.8 cm^3^). Utilizing a higher-capacity battery would allow the pump to operate for more than 7 days on a single charge, without significantly increasing the overall device size.

### 2.4. Delivery Accuracy Characterization in Different Operation Modes

The delivery accuracy of an insulin pump is critical for maintaining stable blood glucose levels in people with diabetes. It is typically assessed by evaluating both single-pulse and average-pulse accuracy over clinically relevant periods.^[33,34]^ Single-pulse accuracy is determined by analyzing individual pulses through multiple measurements, calculating the proportion of pulses that deviate beyond predefined thresholds (±5, ±10, and ±15% of the expected volume), and comparing results across different pumps.^[35]^ Average-pulse accuracy, which offers a more clinically relevant assessment, is typically evaluated by averaging multiple consecutive pulse errors over specific observation periods or targeted insulin delivery volumes.^[36]^ To validate the effectiveness of the proposed magnetic insulin pump, we assess its delivery accuracy and compare it with commercial pumps. The magnetic field profile used in testing is shown in Figure 5B, where the amplitude of the pumping magnetic field is adjusted to achieve different delivery resolutions (see “Supplementary Methods” in the Supporting Information for more details on the accuracy characterization of the magnetic insulin pump).

**Figure 6.**
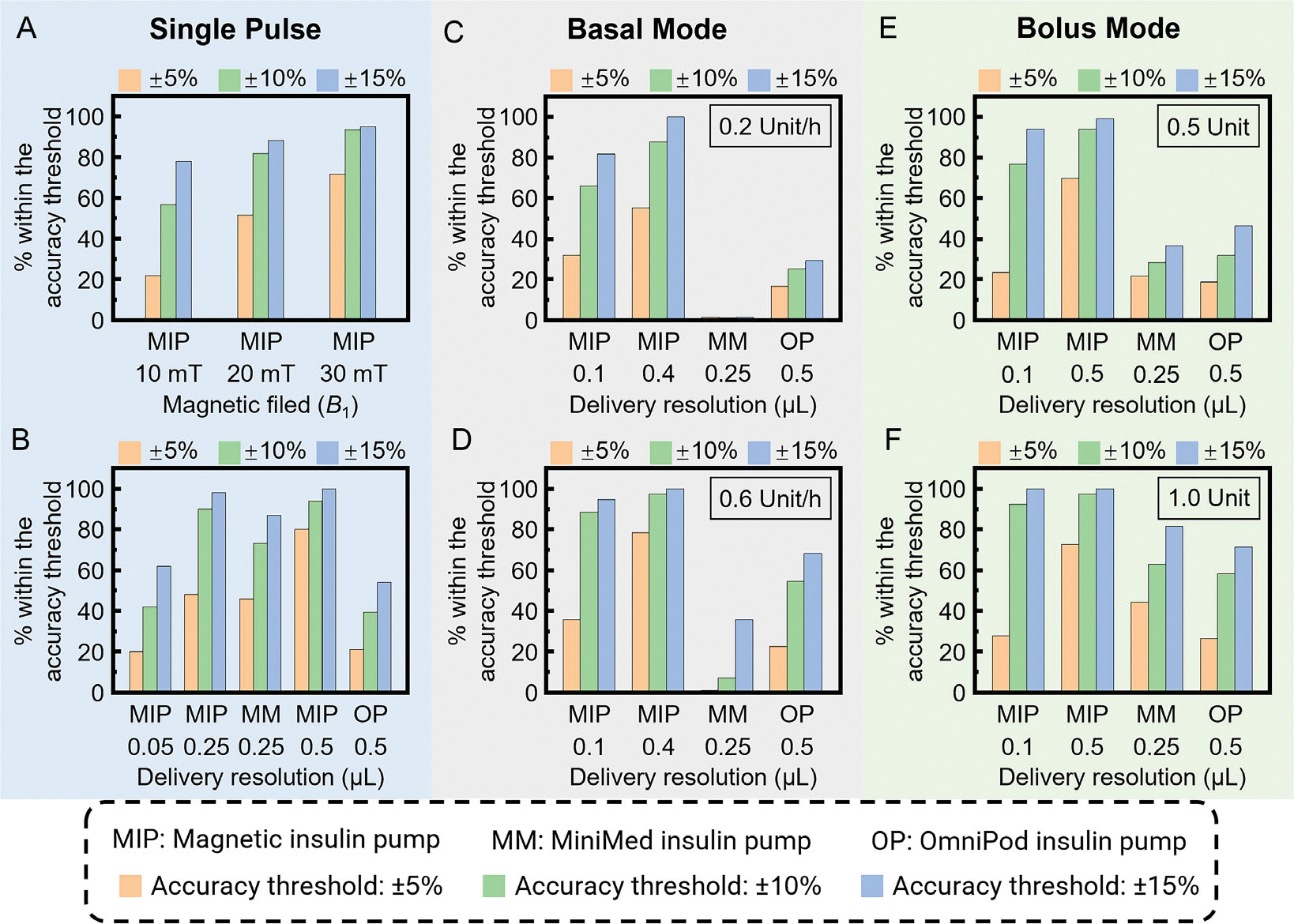
Comparative accuracy study of the magnetic insulin pump and two commercial insulin pumps. The percentage of single pulses within different accuracy thresholds for A) the magnetic insulin pump under different pumping magnetic field amplitudes and B) three insulin pumps with different delivery resolutions. The percentage of basal rates within different accuracy thresholds for three insulin pumps with targeted basal rates of C) 0.2 and D) 0.6 Unit h^−1^. The percentage of bolus doses within different accuracy thresholds for three insulin pumps with targeted basal doses of E) 0.5 Unit and F) 1.0 Unit. Accuracy thresholds are set as ±5, ±10, and ±15%. MIP refers to the magnetic insulin pump. MM refers to the MiniMed insulin pump. OP refers to the OmniPod insulin pump. The accuracy data for two commercial insulin pumps are sourced from the literature.^[35–37]^

First, single-pulse delivery performance is evaluated. Magnetic field amplitudes of 10, 20, and 30 mT are used to assess the single-pulse accuracy. As shown in the measured flow rate results of 10 consecutive pulses (Figure S13, Supporting Information), the pump exhibits high repeatability. To further characterize accuracy, 60 consecutive pulses are analyzed for each amplitude following established test procedures.^[35]^ As illustrated in Figure 6A, increasing the magnetic field amplitude enhances delivery accuracy. At 10 mT, ≈20% of pulses fall within the ±5% accuracy threshold, while at 30 mT, this increases to about 70%. With a ±15% accuracy threshold, around 80% of pulses meet the criterion at 10 mT, and nearly 95% at 30 mT. These results demonstrate the excellent repeatability and precision of the magnetic insulin pump in single-pulse delivery.

The maximum delivery resolutions of Minimed and OmniPod insulin pumps are 0.25 and 0.5 μL, respectively. To compare the single-pulse accuracy of the magnetic insulin pump with these commercial pumps, it is programed to operate at three different delivery resolutions: 0.05, 0.25, and 0.5 μL, corresponding to magnetic fields of 11, 17, and 29 mT, respectively. As shown in Figure 6B, at a 0.05 μL delivery resolution, the magnetic pump’s single-pulse accuracy is comparable to that of the OmniPod pump and slightly lower than that of the MiniMed pump.^[35,37]^ However, when the delivery resolution is 0.25 or 0.5 μL, the magnetic pump outperforms both commercial pumps across all accuracy thresholds (±5, ±10, or ±15%).

Beyond single-pulse accuracy, performance in basal and bolus modes is crucial for people with diabetes. In basal mode, the pump delivers insulin at a steady rate (typically measured in units per hour) to maintain blood glucose levels between meals. In bolus mode, a specific insulin dose is administered to counteract postmeal glucose spikes. Errors in hourly insulin delivery (basal mode) and short-term insulin delivery (bolus mode) are used to evaluate the pump’s average-pulse accuracy. To compare performance, two basal rates (0.2 and 0.6 Unit h^−1^) and two bolus doses (0.5 Unit and 1 Unit) are tested following standardized procedures.^[36]^ The magnetic pump is evaluated at two delivery resolutions (0.1 and 0.4 μL) in basal mode. As seen in Figure 6C,D, its accuracy in basal mode surpasses both commercial pumps, with better performance observed at the 0.4 μL resolution. Figure 6E,F illustrate bolus mode comparisons. When operating at 0.1 μL delivery resolution with ±5% accuracy threshold, the magnetic pump demonstrates accuracy comparable to MiniMed and OmniPod. However, in all other cases, it significantly outperforms both commercial pumps. These results confirm the superior accuracy of the magnetic insulin pump in both basal and bolus modes.

## 3. Conclusion

This work presents a miniaturized, high-accuracy insulin pump driven by a unique magnetic pumping mechanism. Unlike conventional piston-based pumps that require bulky transmission components, the magnetic pump translates the deformation of the magnetic soft actuator directly to insulin delivery. This innovative approach enables both miniaturization and high-resolution insulin delivery. With a total volume of 7.07 cm^3^ and a maximum delivery resolution of 0.01 μL, the proposed magnetic pump significantly outperforms commercial insulin pumps. Our results indicate that the resolution of the pump can be enhanced and tuned by varying the magnetic field’s waveform, amplitude, and application duration, highlighting its operational flexibility. The magnetic pump also demonstrates superior accuracy and repeatability in single-pulse, basal, and bolus modes, surpassing two leading commercial insulin pumps. Its high precision makes it well-suited for clinical diabetes management, with integration into miniaturized automated insulin dosing systems. Additionally, optimizing check valves with lower cracking pressures could further reduce the size and energy consumption of the electromagnetic coil for a more compact pump design. The use of more advanced and higher-precision manufacturing techniques could further optimize the pump’s dimensions and improve the accuracy of insulin delivery.^[38,39]^ With its compact form factor, exceptional accuracy, and adaptability, this magnetic insulin pump is designed to meet the diverse needs of people with diabetes while enhancing their overall user experience.

## 4. Experimental Section

### Fabrication of the Magnetic Insulin Pump:

The magnetic insulin pump was designed as a two-part system assembled via magnetic attraction, as shown in Figure 2D. This system consists of a base part and a replaceable part. The base part houses the battery, driver board, and electromagnetic coil, while the replaceable part contains the reservoir, chamber, and magnetic soft actuator. The casings of both parts were fabricated using Rigid resin by a stereolithography resin 3D printer (Form 3+, Formlabs Inc., USA). Magnetic-assisted assembly was enabled by embedding six miniature magnets (dimension: *φ*1 mm × 0.5 mm) on the contact surfaces of both parts (Figure S1, Supporting Information). In the replaceable part, the installation hole diameter was 0.2 mm smaller than the outer diameter of the check valve (CCPI2580004S, The Lee Company, USA) to ensure a tight fit. The membrane of the magnetic soft actuator (dimension: 10.1 × 7.4 × 0.25 mm) was fabricated by spin-coating silicone rubber precursor (Ecoflex 00–30, Smooth-On, Inc., USA) at 400 rpm for 30 s, followed by curing at 60 °C for 10 min. After curing, the membrane was glued on one side to an NdFeB magnet (N52, 4.0 × 2.3 × 1.4 mm) and on the other side to the chamber using Sil-Poxy adhesive (Smooth-On, Inc., USA). In the base part, all components were glue-assembled.

### Magnetic Actuation of the Magnetic Insulin Pump:

To evaluate pump performance, magnetic fields with various profiles were programed and generated using the electromagnetic actuation system. Each magnetic field profile could be divided into three states, namely the pumping state, the refilling state, and the recovery state. A switch in the driver board was used to initiate the preprogrammed magnetic field profile of the magnetic insulin pump. For all experimental results in Figure 5, each magnetic field profile was tested in five consecutive trials, and the reported delivery volume was the average of these five experiments.

### Study Approval:

The on-body demonstrations of insulin pumps with glucose sensors in Figure 2E were carried out in full compliance with local laws and ethical guidelines. Written informed consent was obtained from the involved participant.

## Supplementary Material

Supplement

Supporting Information

Supporting Information is available from the Wiley Online Library or from the author.

## Data Availability

The data that support the findings of this study are available in the supplementary material of this article.
