# Supplementary material for: A Mini-Patch Magnetic Insulin Pump for Enhanced Delivery Resolution and Accuracy
Source: Adv Intell Syst. Author manuscript; Available in PMC 2026 Feb 21. (PMC12922623; doi:10.1002/aisy.202500459)
Supplement: Supplement [file NIHMS2139671-supplement-Supplement.zip › Supporting information.pdf]

Supporting Information

A Mini-Patch Magnetic Insulin Pump for Enhanced Delivery Resolution and Accuracy

*Qiji Ze, Shuhao Huang, Yilong Chang, Jize Dai, Rayhan A. Lal, Ruike Renee Zhao**

Supplementary Methods

The electromagnetic actuation system of the magnetic insulin pump. The precisely controlled magnetic field required for the magnetic insulin pump is generated by a customized electromagnetic actuation system located in the base part of the pump. This system comprises a battery, a driver board, and an electromagnetic coil. The schematic diagram of the system is provided in Figure S2A. The system uses a single-cell lithium-ion battery (Dimension: 20 mm × 10 mm × 4 mm) with a capacity of 120 mAh. The driver board features a DC/DC converter chip (TPS61092RSAR) that boosts the battery's output voltage from 3.7 V to 5 V, supplying power to both the microcontroller unit (MCU, STC8H3K48S4) and the H-Bridge driver chip (DRV8212). The H-Bridge driver operates in PWM (pulse-width modulation) mode and can deliver up to 2 A of current to the electromagnetic coil. By programming the PWM control signal, both the polarity and amplitude of the current through the coil can be precisely controlled. A Hall-effect current sensor (CC6920SO-5A) is used to monitor the current in real-time and provides feedback to the MCU, enabling accurate closed-loop current regulation. Additionally, a switch connected to the MCU allows users to initiate the pre-programmed operation of the magnetic insulin pump, as demonstrated in Video S1. A prototype of the driver board is shown in Figure S2B.

Optimization of the electromagnetic coil and magnetic soft actuator. To achieve optimal delivery efficiency, finite element analysis (FEA) is conducted using COMSOL Multiphysics software (COMSOL, Inc., Sweden) to optimize the design parameters of both the electromagnetic coil and the magnetic soft actuator within the magnetic insulin pump. The predefined dimensions of the coil and the membrane are illustrated in Figure S6.

In the electromagnetic FEA model, the current flowing through the coil cross-section is determined by the diameter of the selected copper wire. Given the resistivity of copper (1.72×10^{-8} Ω·m), the number of turns and total resistance of the coil are calculated for various wire diameters: 0.17 mm, 0.23 mm, 0.27 mm, 0.35 mm, 0.39 mm, 0.45 mm, and 0.52 mm. Additionally, considering the battery's discharge capability, the maximum allowable voltage and current are limited to 5 V and 2 A, respectively. As shown in Figure 4A, the electromagnetic

coil generates the strongest magnetic field at the center of the magnet when the copper wire diameter is 0.35 mm, indicating this configuration offers the best magnetic field output. All results on the magnetic field distribution within the coil in Figure 4B and Figure S7 are exported from the electromagnetic FEA model with the copper wire diameter of 0.35 mm and the magnetic field B_1 of 20 mT.

In the magneto-mechanical FEA model, the dimensions of the membrane and the magnet are set to 6.5 mm × 4.0 mm × 0.25 mm and 4.0 mm × 2.3 mm × 1.4 mm, respectively. The Young's modulus is set to 50 kPa for the membrane and 150 GPa for the magnet. The remanent magnetization of the magnet is defined as 1130 kA/m. Under boundary conditions where all four edges of the membrane are fixed, simulations are performed to evaluate the deformation of the magnetic soft actuator when a magnetic field of 20 mT is applied. By varying the distance between the magnet and the right short edge of the membrane, the resulting deformations and corresponding volume changes are calculated and presented in Figure 4C. The simulation results indicate that the volume change caused by membrane deformation decreases as the magnet is positioned further from the membrane's right short edge.

Experimental and quantitative verification of the magnetic torque-based actuation mechanism. The magnetic field distribution on the *X*-*Z* plane in Figure S7 reveals the presence of a magnetic field gradient along the *Z*-axis, which may induce magnetic force on the magnet of the magnetic soft actuator. To verify the magnetic torque-based actuation mechanism of the magnetic insulin pump, we first fabricate a simplified model without the chamber and reservoir to facilitate direct observation of the magnet's motion, as shown in Figure S3A. In this model, the spatial arrangement of the magnetic soft actuator remains identical to that in the actual pump. During the experiment, the applied magnetic field amplitudes for the pumping and refilling states are set to 20 mT and -10 mT, respectively. As shown in Figure S3B, the magnet undergoes clockwise rotation under a positive magnetic field and counterclockwise rotation under a negative field. This bidirectional rigid-body rotation provides direct experimental evidence supporting the torque-induced actuation mechanism.

To further quantify the contributions of magnetic force and torque, FEA simulations are conducted under the pumping condition (B_1 = 20 mT), as shown in Figure S4. As illustrated in Figure S4B, the magnetic force components along the *Y*- and *Z*-axes remain negligible (approaching zero) throughout the magnet's rotation, while a constant force of approximately 10 mN is maintained along the *X*-axis. However, due to the constraint imposed by the magnet–membrane interface, displacement along the *X*-axis does not result in membrane deformation.

These results confirm that magnetic force plays a minimal role in the actuation process. In contrast, the magnetic torque remains consistently high during the rotation (Figure S4C), providing strong quantitative validation that the proposed magnetic insulin pump operates primarily through magnetic torque-induced rotation of the embedded magnet.

Details on magnetic actuation experiments of the magnetic insulin pump. The delivery performance of the magnetic insulin pump is directly influenced by the deformation of the magnetic soft actuator, which is controlled by the applied magnetic field. To evaluate pump performance, magnetic fields with various profiles are programmed and generated using the electromagnetic actuation system. Figure 5A illustrates the experimental setup. A medical infusion set is connected to a piece of pork skin to simulate real-world usage of an insulin pump. A 0.37 mm-diameter, 6 mm-long needle is inserted through the skin into the underlying fat layer to ensure effective fluid delivery. The pump, flow meter, and infusion set are connected via silicone tubing. Flow rates are directly measured using a high-precision flow meter (SLI-0430, Sensirion, Switzerland), which has a measurement range of 0-120 μL/min and a sampling interval of 0.1 s. The total delivery volume is calculated by integrating the flow rate data through the flow meter's GUI software.

In this study, only the positive magnetic field associated with the pump's pumping state is varied. The magnetic field settings for the refilling and recovery states remain constant throughout the experiments. Specifically, during the refilling state, a negative magnetic field of -10 mT is applied for 1 s, while in the recovery state, no magnetic field is applied, and a fixed waiting period of 20 s is maintained. In the pumping state, square-wave and trapezoidal-wave magnetic fields with different amplitudes, durations, and ramp-up times are applied to investigate their effect on the pump's flow rate. Experimental results are presented in Figure 5, Figure S9, Figure S10, and Figure S11. Notably, each magnetic field profile is tested in five consecutive trials, and the reported delivery volume is the average of these five experiments.

Details on the accuracy characterization of the magnetic insulin pump. To evaluate the performance of the proposed magnetic insulin pump, extensive repeated experiments are conducted under three operational modes: single-pulse mode, basal mode, and bolus mode. The experimental results are benchmarked against those of two commercial insulin pumps reported in the literature. All tests use the magnetic field profile illustrated in Figure 5B. The pump's delivery volume is regulated solely by adjusting the amplitude of the pumping magnetic field. The amplitude and duration of the magnetic field during the refilling state are fixed at -10 mT and 1 s, respectively. The waiting time between pulses is set to 20 s for both the single-pulse

and bolus modes. In basal mode, the waiting time is determined by the required pulse frequency to meet the target delivery rate. For the experiments corresponding to Figure 6A, three specific magnetic field amplitudes (10 mT, 20 mT, and 30 mT) are applied. The amplitudes used in the other subfigures of Figure 6 are selected based on the desired delivery resolution and the flow rate characterization results shown in Figure 5D.

In single-pulse mode, each magnetic field profile is repeated 60 times consecutively to assess repeatability and consistency. For the basal and bolus modes, it is assumed that U-500 insulin is used, where 1 unit corresponds to an accumulated delivery volume of 2 μL. In basal mode, two target delivery rates (0.2 Units/hour and 0.6 Units/hour) and two delivery resolutions (0.1 μL and 0.4 μL) are tested. The magnetic insulin pump adjusts the number of pulses per hour to achieve each specified basal rate. Each condition shown in Figure 6C and Figure 6D is continuously tested over a 22-hour period. In bolus mode, two target bolus doses (0.5 Units and 1.0 Unit) and two delivery resolutions (0.1 μL and 0.5 μL) are evaluated. The pump delivers the required number of consecutive pulses to match each bolus dose. Each condition illustrated in Figure 6E and Figure 6F is tested in 25 consecutive trials.

Thermal safety evaluation of the magnetic insulin pump. To evaluate whether the heat generated by the electromagnetic coil poses any thermal safety concerns for on-body use, we measure the surface temperature of the magnetic insulin pump under different pumping magnetic field amplitudes (10 mT, 20 mT, and 30 mT). The experimental setup is shown in Figure S14A, with an ambient temperature of 20°C. The magnetic field profiles used in the test are shown in Figure 5B. For each magnetic field amplitude, 20 consecutive actuation cycles are applied. Figure S14B presents the surface temperature evolution of the device over these cycles. The results show that even at a pumping magnetic field amplitude of 30 mT, the maximum surface temperature only reaches 38.2°C, which is close to human body temperature. When the amplitude is reduced to 20 mT, the coil stabilizes at approximately 29°C after 10 cycles. Furthermore, considering the energy consumption characteristics of the magnetic insulin pump (Figure S12), the duration of the pumping magnetic field is expected to be relatively short in practical applications. As a result, significantly less heat would be generated under normal operating conditions. In addition, the pump does not need to operate continuously for tens of cycles, further supporting its thermal safety for on-body use.

Supplementary Figures and Figure Captions

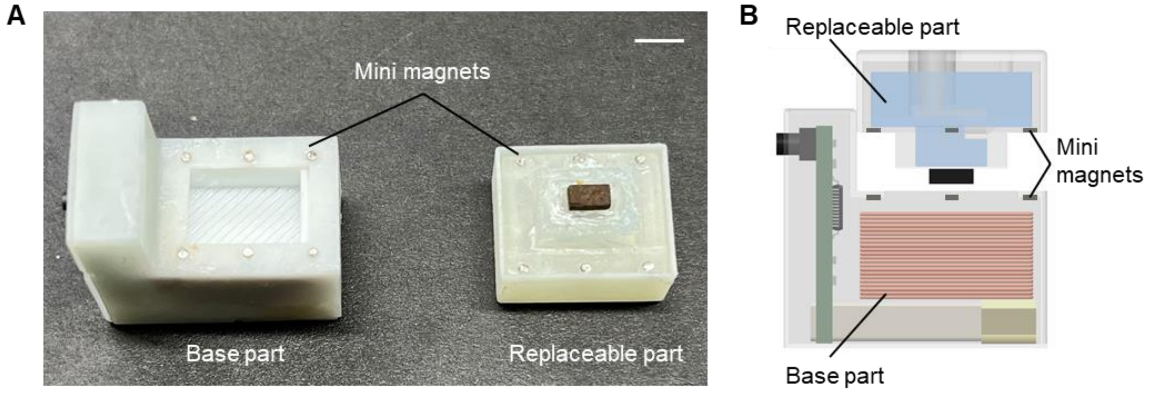

Figure S1. Schematic of the magnetic-assisted assembly. (A) Mini magnets on the contact surfaces of the base part and the replaceable part. Scale bar: 5 mm. (B) Assembly model.

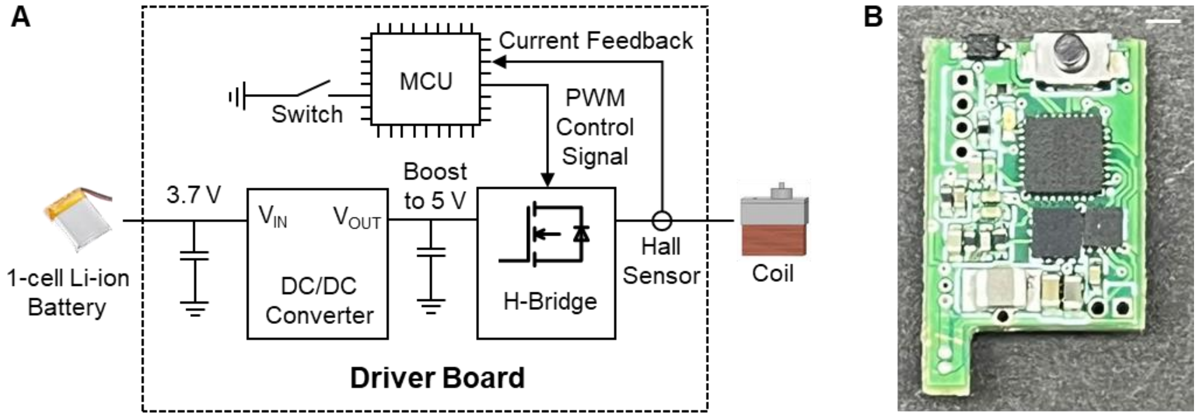

Figure S2. The electromagnetic actuation system of the magnetic insulin pump. (A) Schematic of the electromagnetic actuation system. (B) Prototype of the driver board. Scale bar: 2 mm.

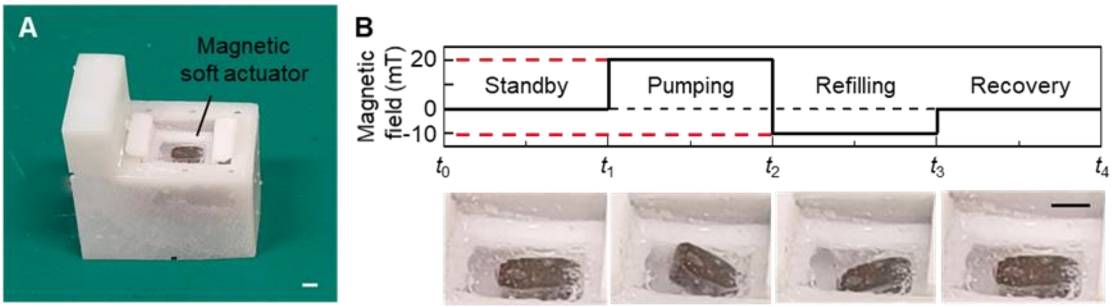

Figure S3. Experimental verification of the magnetic actuation mechanism. (A) The magnetic insulin pump prototype without the chamber and reservoir. (B) Photos of the magnetic soft actuator in different states. Scale bars: 2 mm.

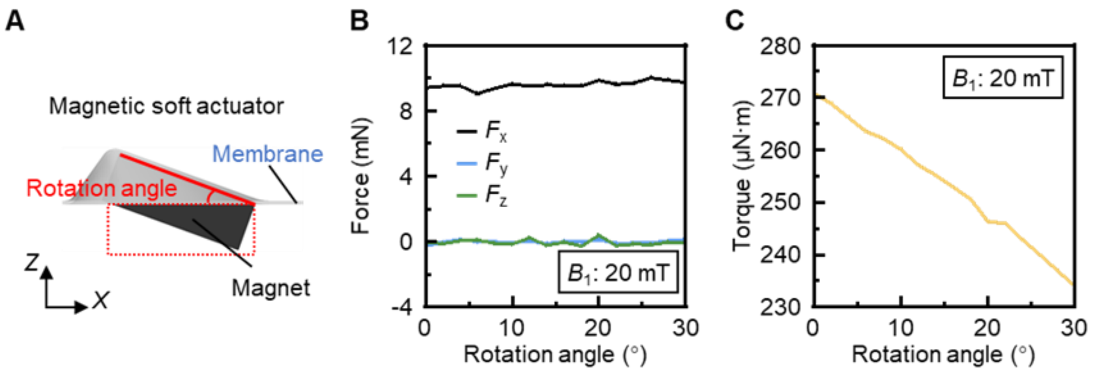

Figure S4. Simulated results of the induced magnetic force and magnetic torque in the pumping state of the magnetic insulin pump. (A) Schematic of the rigid-body rotation of the magnet. (B) The magnetic force induced in the magnet along different axes. (C) The magnetic torque induced in the magnet in the clockwise direction.

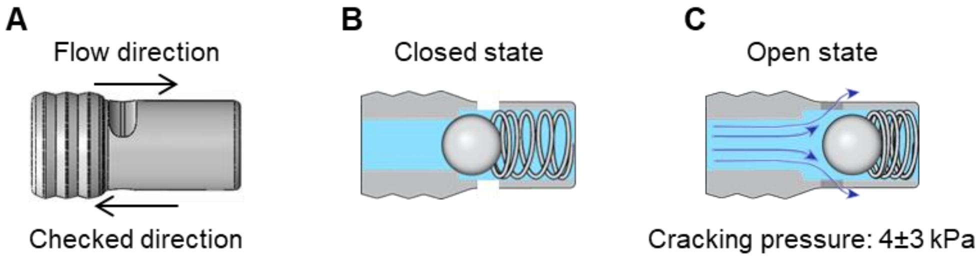

Figure S5. The one-way check valve. (A) The schematic of the check valve. (B) The closed state and (C) the open state of the check valve. The cracking pressure of the check valve is 4 kPa.

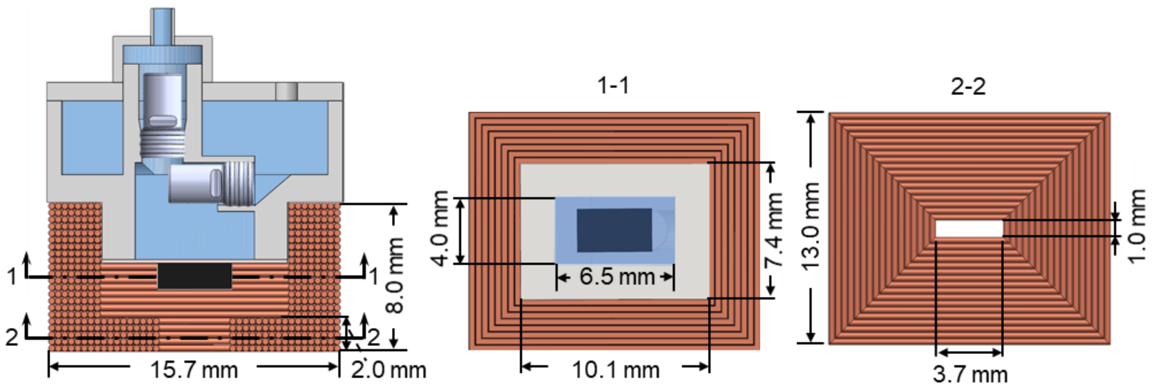

Figure S6. The preset dimensions of the electromagnetic coil and the effective membrane region.

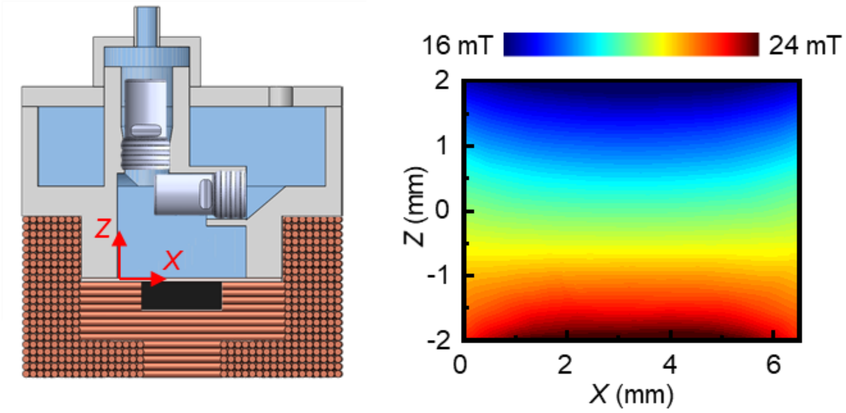

Figure S7. Simulated magnetic field distribution on the plane which is perpendicular to the membrane.

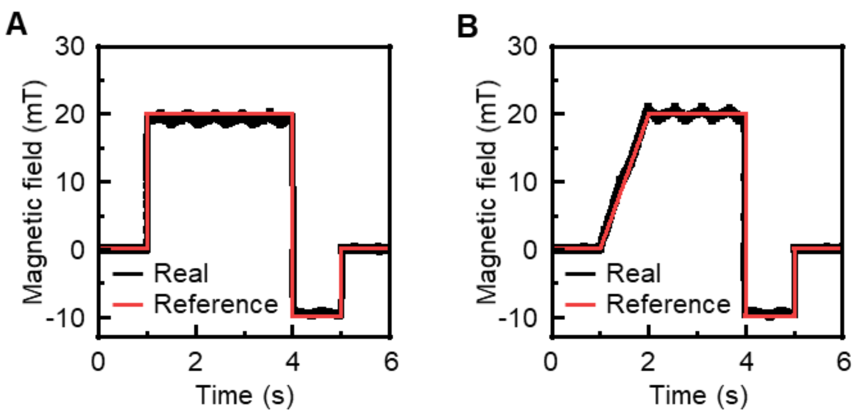

Figure S8. The real magnetic field waveforms with (A) square-wave and (B) trapezoidal-wave pumping magnetic fields.

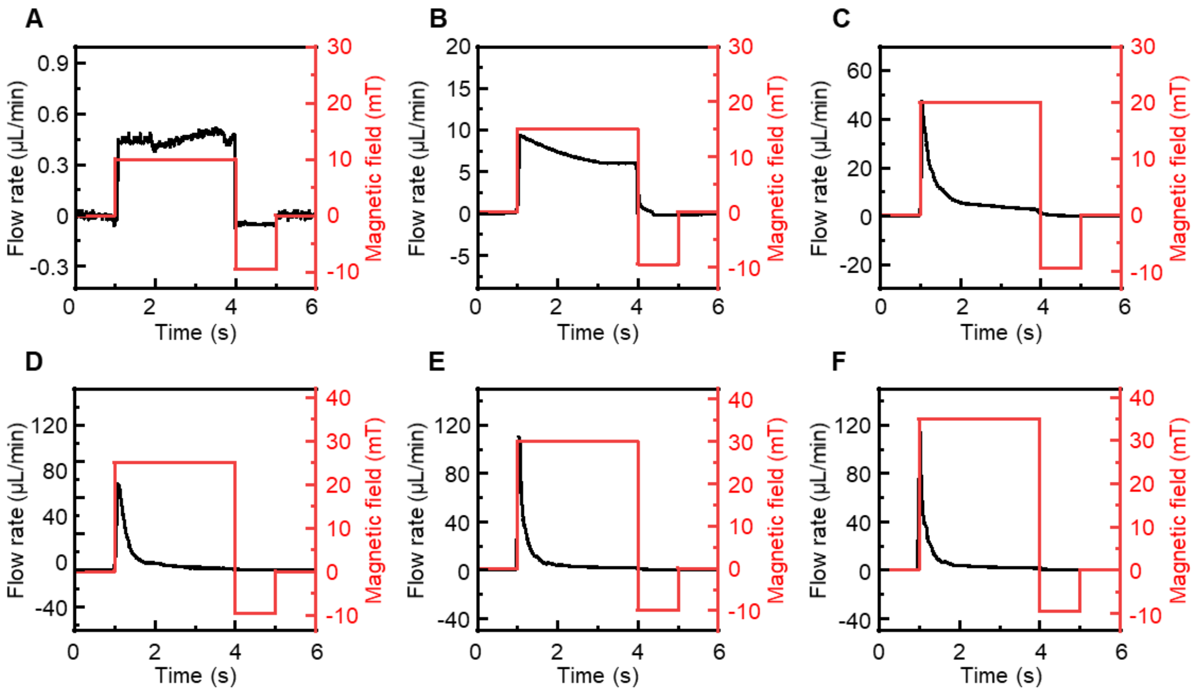

Figure S9. The extrusion flow rate of the pump when square-wave magnetic fields with different amplitudes are applied in the pumping state. The amplitudes of pumping magnetic fields are (A) 10 mT, (B) 15 mT, (C) 20 mT, (D) 25 mT, (E) 30 mT, and (F) 35 mT, respectively.

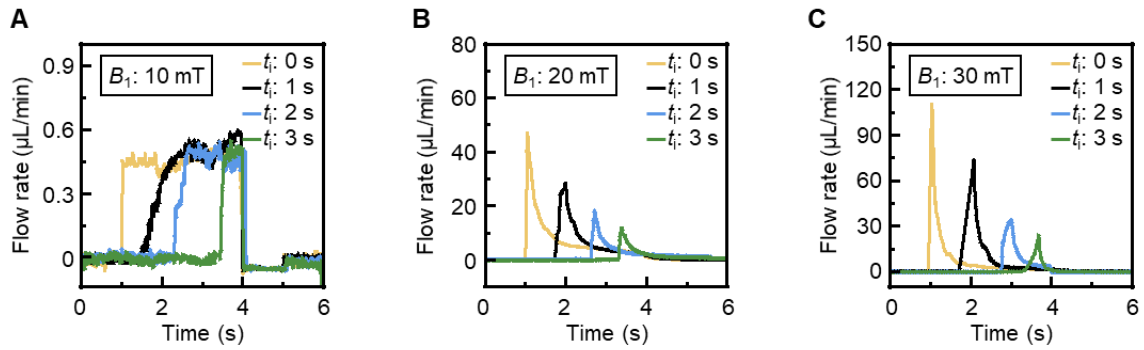

Figure S10. The extrusion flow rate of the pump when trapezoidal-wave magnetic fields with different ramp-up times are applied in the pumping state. The amplitudes of pumping magnetic fields are (A) 10 mT, (B) 20 mT, and (C) 30 mT, respectively.

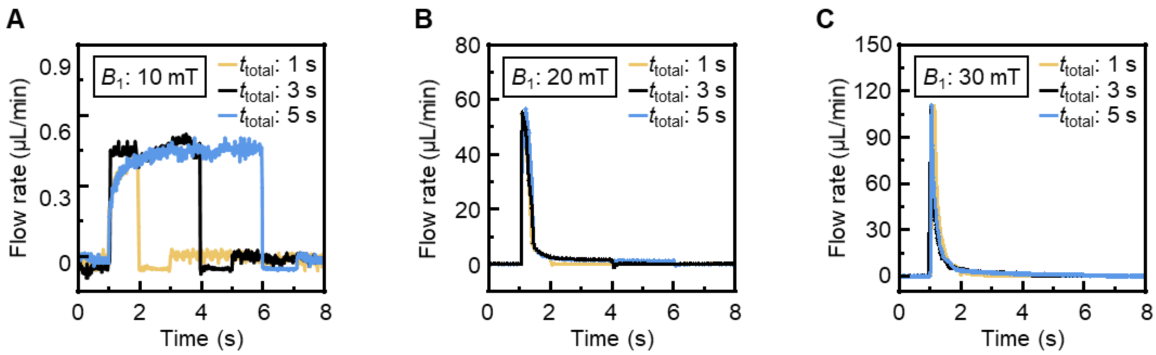

Figure S11. The extrusion flow rate of the pump when square-wave magnetic fields with different durations are applied in the pumping state. The amplitudes of pumping magnetic fields are (A) 10 mT, (B) 20 mT, and (C) 30 mT, respectively.

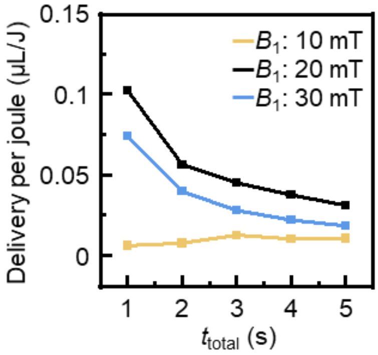

Figure S12. Influence of the pumping magnetic field duration and amplitude on the energy consumption.

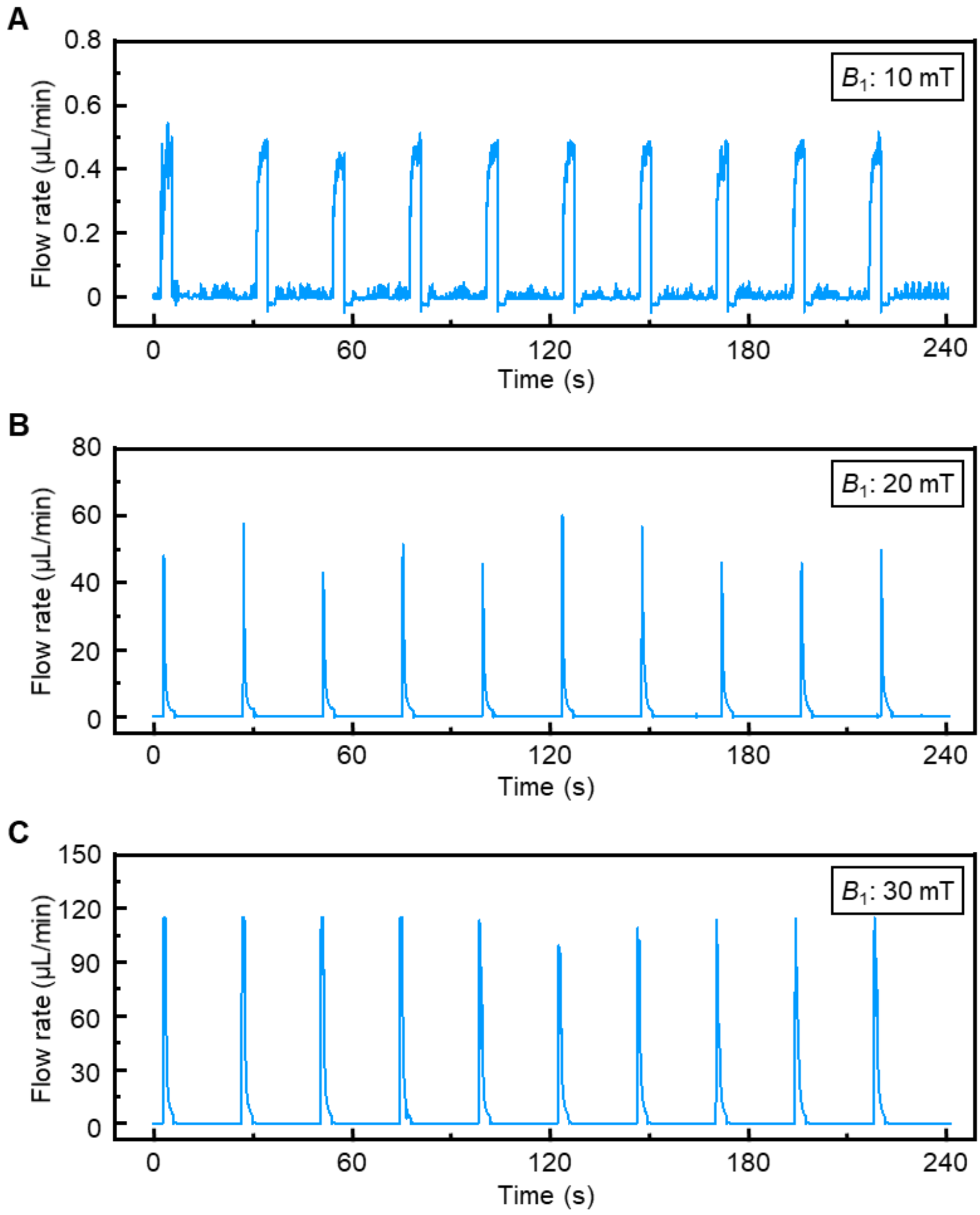

Figure S13. The extrusion flow rate results of 10 consecutive pulses. All experiments use the magnetic field profile shown in Figure 5B. The amplitudes of pumping magnetic fields are (A) 10 mT, (B) 20 mT, and (C) 30 mT, respectively.

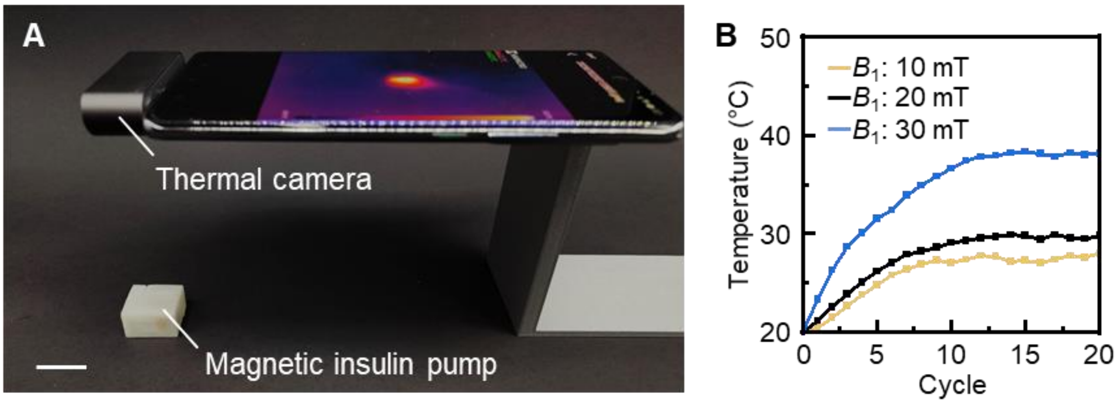

Figure S14. Thermal safety evaluation of the magnetic insulin pump. (A) Experimental setup for surface temperature measurement. Scale bar: 20 mm. (B) Surface temperature evolution over 20 actuation cycles with different pumping magnetic field amplitudes.

Supplementary Tables and Table Captions

Table S1. Comparison between the magnetic insulin pump and two commercial insulin pump products.

	Dimension	Volume	Weight	Delivery resolution	Reservoir size
Magnetic insulin pump (This work)	23.5 mm × 21.5 mm × 14.0 mm	7.07 cm^3	10.5 g	0.01 µL	0.8 mL
MiniMed 530G insulin pump	94.0 mm × 50.8 mm × 20.3 mm	84.74 cm^3	104 g	0.25 µL	3 mL
OmniPod insulin pump	52.1 mm × 38.9 mm × 14.5 mm	29.39 cm^3	26 g	0.50 µL	2 mL

Supplementary Video Captions

Video S1: Pump design and controllable liquid delivery.

Video S2: Working mechanism and performance characterization.
